# Epidemiology and Antifungal Susceptibility Profile of *Aspergillus* Species: Comparison between Environmental and Clinical Isolates from Patients with Hematologic Malignancies

**DOI:** 10.1128/JCM.02023-18

**Published:** 2019-06-25

**Authors:** Sung-Yeon Cho, Dong-Gun Lee, Won-Bok Kim, Hye-Sun Chun, Chulmin Park, Jun-Pyo Myong, Yeon-Joon Park, Jae-Ki Choi, Hyo-Jin Lee, Si-Hyun Kim, Sun Hee Park, Su-Mi Choi, Jung-Hyun Choi, Jin-Hong Yoo

**Affiliations:** a Division of Infectious Diseases, Department of Internal Medicine, College of Medicine, The Catholic University of Korea, Seoul, Republic of Korea; b Vaccine Bio Research Institute, The Catholic University of Korea, Seoul, Republic of Korea; c Catholic Hematology Hospital, Seoul, Republic of Korea; d Occupational and Environmental Medicine, College of Medicine, The Catholic University of Korea, Seoul, Republic of Korea; e Department of Laboratory Medicine, College of Medicine, The Catholic University of Korea, Seoul, Republic of Korea; Carter BloodCare & Baylor University Medical Center

**Keywords:** *Aspergillus*, azoles, drug resistance mechanisms, environmental microbiology, hematologic diseases

## Abstract

Global data on the epidemiology and susceptibility of *Aspergillus* are crucial in the management of invasive aspergillosis. Here, we aimed to determine the characteristics of clinical and environmental *Aspergillus* isolates, focusing mainly on hematologic malignancy patients. We prospectively collected all consecutive cases and clinical isolates of culture-positive proven/probable invasive aspergillosis patients from January 2016 to April 2018 and sampled the air inside and outside the hospital. Cryptic species-level identification of *Aspergillus*, antifungal susceptibilities, and *cyp51* gene sequencing were performed, and clinical data were analyzed. This study was conducted as part of the Catholic Hematology Hospital Fungi Epidemiology (CAFÉ) study. A total of 207 proven/probable invasive aspergillosis and 102 clinical and 129 environmental *Aspergillus* isolates were included in this analysis. The incidence of proven/probable invasive aspergillosis was 1.3 cases/1,000 patient-days during the study period. Cryptic *Aspergillus* species accounted for 33.8%, with no differences in proportions between the clinical and environmental isolates. Section *Nigri* presented a high proportion (70.5%) of cryptic species, mainly from A. tubingensis and *A. awamori*: the former being dominant in environmental samples, and the latter being more common in clinical isolates (*P* < 0.001). Of 91 A. fumigatus isolates, azole-resistant A. fumigatus was found in 5.3% of all A. fumigatus isolates. Three isolates presented the TR_34_/L98H mutation of the *cyp51A* gene. Patients with invasive aspergillosis caused by azole-resistant A. fumigatus showed 100% all-cause mortality at 100 days. This study demonstrates the significant portion of cryptic *Aspergillus* species and clinical implications of azole resistance and underscores the comparison between clinical and environmental isolates.

## Abstract (precis)

Global data on the epidemiology and susceptibility of *Aspergillus* are crucial in the management of invasive aspergillosis. Here, we aimed to determine the characteristics of clinical and environmental *Aspergillus* isolates, focusing mainly on hematologic malignancy patients.

## INTRODUCTION

Invasive aspergillosis (IA) is a major infectious complication that develops after intensive chemotherapy or hematopoietic stem cell transplantation (HSCT) for hematologic malignancies. Although there is little controversy that the prophylaxis and treatment of IA have advanced over the last decade, the morbidity and mortality of IA remain high. Voriconazole is the drug of choice for treating IA, and isavuconazole has also become a first-line targeted therapy according to the recent international guideline (1–3). Posaconazole is recommended for primary antifungal prophylaxis during remission-induction chemotherapy, immunosuppressive therapy for graft-versus-host diseases after HSCT, and salvage therapy of refractory IA (1–5). However, concerns about changing epidemiology, including azole resistance or IA caused by cryptic *Aspergillus* species, are rising.

In epidemiological surveys in Spain and the United States, cryptic species accounted for 10 to 15% of all *Aspergillus* isolates (6–8). Cryptic species refers sibling species that are difficult to be distinguished by morphological identification and which exhibit distinctive molecular and phenotypic characteristics. The clinical significance of these cryptic species is that they can exhibit intrinsic resistance, with an *in vitro* resistance rate of about 40% against at least one antifungal agent (6, 9). Moreover, there has been an increase in the reports of azole-resistant *Aspergillus* species in recent years (10). Two possible routes of invasive diseases caused by azole-resistant *Aspergillus* are known: the environmental route (associated with fungicide use) or the patient route (resulting from long-term azole therapy) (11, 12). The main route of resistance development is considered to be through agricultural fungicide use (12). Since the azole resistance rate varies by region, global epidemiological data are needed. Furthermore, the characteristics of environmental and clinical isolates in azole-resistant *Aspergillus* must be compared for an integrated understanding of antifungal resistance. To establish an accurate epidemiological cutoff value (ECV) and the future breakpoint for *Aspergillus* species, more epidemiological data are needed, together with clinical information, including the treatment outcome of IA caused by azole-resistant strains (13, 14).

Here, we aimed to identify the epidemiology of *Aspergillus* species, including cryptic species distribution, susceptibility profiles, and phylogenetic analysis of clinical and environmental *Aspergillus* isolates. We also evaluated the treatment course and clinical outcome of IA caused by azole-resistant *Aspergillus* species and sought to investigate the relationship or differences between clinical and environmental isolates in this study.

## MATERIALS AND METHODS

### Study design and hospital setting.

We prospectively collected all consecutive cases of culture-positive proven or probable IA and *Aspergillus* clinical isolates from patients with hematologic malignancies from January 2016 to April 2018 at Catholic Hematology Hospital of Seoul St. Mary’s Hospital, Seoul, South Korea. At this university-affiliated, tertiary hospital, over 500 HSCTs are performed annually. *Aspergillus* clinical isolates obtained from a sterile site or the lower respiratory tract and demonstrating clinical significance as previously described were included in this study (15, 16). IA was defined according to the revised deﬁnition of invasive fungal disease (IFD) from the European Organization for the Research and Treatment of Cancer/Mycoses Study Group (EORTC/MSG) (16). Clinical isolates were classified as a pathogen or colonization. Data on baseline characteristics, IFD results (clinical, microbiologic, laboratory, and radiological), antifungal use, and patient outcome were collected. This study was conducted as part of the Catholic Hematology Hospital Fungi Epidemiology (CAFÉ) study. The Institutional Review Board of Seoul St. Mary’s Hospital approved the research protocol of the study (KC16SISI0307).

### Sampling of indoor and outdoor air.

Air was sampled at four different locations of the hospital, including one outdoor and three indoor locations: (i) outside the hospital main building, (ii) the lounge of the general ward (GW), (iii) the cleanroom hallway of a chemotherapy unit, and (iv) a patient’s room in the cleanroom of the chemotherapy unit. The location of the hospital is one of the representative districts of Seoul, within a mile radius of high-rise buildings, two parks, and riverside. The hospital building has a heating, ventilation, and air conditioning system installed. The chemotherapy unit is a 10,000-class cleanroom with a high-efficiency particulate air filtration system and positive pressure. Air sampling at each location was conducted bimonthly at the same time (between 1400 and 1530 h) from May 2017 to April 2018, considering a seasonal variation. Air sampling with a KAS-110 air sampler (Kemic Corp., Seongnam, South Korea; Ministry of Environment approval number IASM-2012-2) with a collection velocity of 16 liters/min was used for 15 min three times, with 20-min intervals to factor in air circulation. Sabouraud dextrose agar (SDA) plates were used as the impactor substrates and incubated at 35°C for 4 to 5 days (17). Fungal CFU were counted, and spots of airborne fungi were subcultured in a new SDA plate. Observed fungal colony count was calculated as the average CFU of the three plates obtained from each location.

### Identification of fungi and phylogenetic analysis.

For the molecular identification of *Aspergillus* species, we performed internal transcribed spacer (ITS) sequencing and PCR of the β-tubulin gene (*benA*). The entire ITS region was amplified using the primers ITS1-F_KYO2 (5′-TAGAGGAAGTAAAAGTCGTAA-3′) and ITS4 (5′-TCCTCCGCTTATTGATATGC-3′) (18). β-Tubulin PCR was performed using the primers bt2a (5′-GGTAACCAAATCGGTGCTGCTTTC-3′) and bt2b (5′-ACCCTCAGTGTAGTGACCCTTGGC-3′) (18). The promoter region of *cyp51A* in A. fumigatus isolates was amplified using the primers AFTR-F (5′-TAATCGCAGCACCACTTCAG-3′) and AFTR-R (5′-GCCTAGGACAAGGACGAATG-3′) (19). The whole *cyp51A* gene of A. fumigatus was amplified using the primers wholeF (5′-TAATCGCAGCACCACTTCAG-3′) and wholeR (5′-CCGATCACACCAAATCCTTT-3′), designed in this study; the thermal profile included an initial denaturation step at 94°C for 5 min; 30 cycles of denaturation at 94°C for 30 s, annealing at 59°C for 30 s, and elongation at 72°C for 30 s; and a final extension at 72°C for 10 min. Next, mutations were profiled by the comparison of their amino acid sequences to those of azole-susceptible reference strains (AF293, GenBank no. CM000172; MYA3626, KX159723). The *cyp51A* gene of the section *Nigri* was also analyzed by comparison to those of NRRL3357 (wild-type [WT] strain), as previously described (20). The mutation profiles of *cyp51* genes (*cyp51A*, *cyp51B*, and *cyp51C*) in A. flavus strains were analyzed by comparison to those of NRRL3357 (WT strain), as previously described (21). Phylogenetic analysis was performed using the maximum-likelihood method based on the Tamura-Nei model of MEGA7 (22). Control sequences used in this study are shown in in the supplemental material.

### Antifungal susceptibility testing and interpretation.

MICs were determined using the broth dilution method, as recommended by the Clinical and Laboratory Standards Institute (CLSI) M38-A2 (23). Itraconazole (Sigma-Aldrich, St. Louis, MO), voriconazole (Pfizer, Inc., New York, NY), posaconazole (Sigma-Aldrich), and amphotericin B (AMB) solubilized powder (Sigma-Aldrich) were used for MICs. Minimal effective concentrations (MECs) were determined by microscopic examination of the microdilution plates at 24 and 48 h. The least concentration of caspofungin (Merck & Co., Inc., Kenilworth, NJ), anidulafungin (Pfizer), or micafungin (Astellas Pharma, Inc., Tokyo, Japan) causing abnormal hyphal growth with short abundant branching was defined as the MEC. The reference strain Candida parapsilosis (ATCC 22019) was used as the quality control isolate for each antifungal susceptibility test. We defined azole resistance according to published reports and the ECV proposed by CLSI to evaluate noncryptic *Aspergillus* species (24, 25); therefore, the breakpoints were applied as presented in Table SA in the supplemental material. For cryptic species, MIC distribution was presented without applying the ECV of each section.

### Statistical analysis.

Statistical analysis was performed using SPSS software, version 24.0 (SPSS Korea, Seoul, South Korea). Chi-square analysis was used to compare categorical variables. Spearman rank correlation coefficient was used for correlation analysis. A two-tailed *P* value of ≤0.05 was considered statistically significant.

### Accession number(s).

The GenBank accession numbers for *benA* sequences of *Aspergillus* isolates determined in this study are MH781272 to MH781343. The GenBank accession numbers for *cyp51A* sequences of *Aspergillus* isolates belonging to the section *Nigri* and A. fumigatus are MH781344 to MH781399.

## RESULTS

### Incidence of IA and *Aspergillus* clinical and environmental isolates.

**FIG 1:**
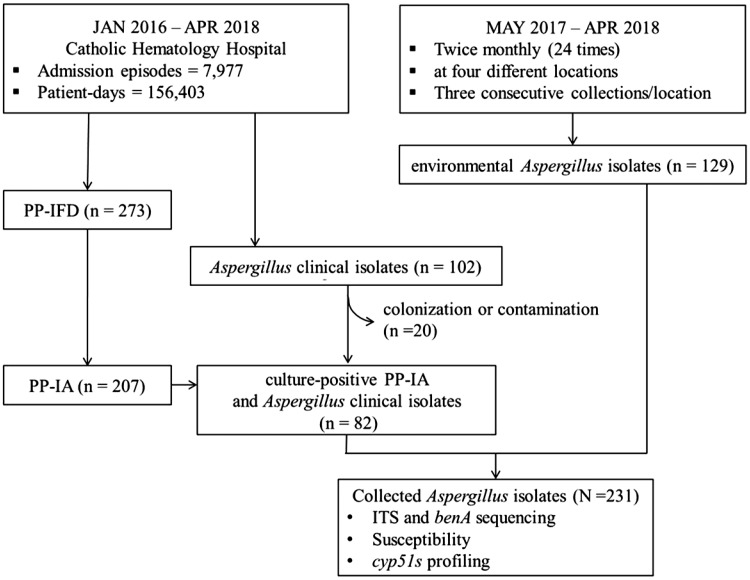
Cases and flow of the study. PP-IA, proven/probable invasive aspergillosis; PP-IFD, proven/probable invasive fungal diseases.

**FIG 2:**
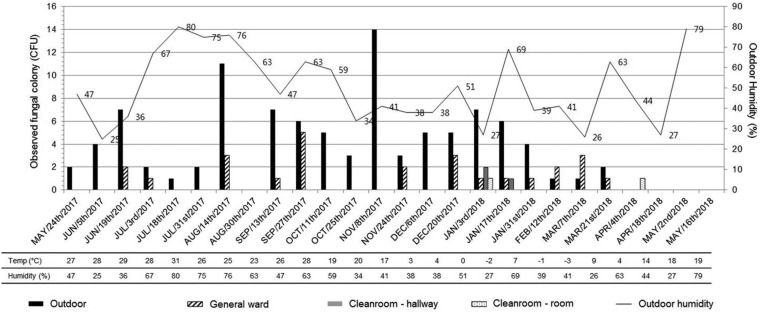
Variations in *Aspergillus* colony counts from collected air at each location. Temp, temperature.

During the study period, 273 proven/probable IFDs and 207 proven/probable IAs were identified from 7,977 admission episodes. The incidence of proven/probable IA was 1.3 cases/1,000 patient-days and 2.6 cases/100 admissions in Catholic Hematology Hospital during the study period. The cases and flow of this study are presented in Fig. 1. Of the 108 collected *Aspergillus* clinical isolates, we excluded six isolates cultured from the same patient within 7 days. Among 102 consecutive *Aspergillus* clinical isolates, 82 were considered pathogens of IA. Furthermore, 129 environmental *Aspergillus* isolates were identified from 288 air samples collected throughout the year, which were also included in this study. Of the 129 environmental isolates, 98 (76.0%) and 26 (20.2%) were from the outdoor air and the air collected at the lounge of the GW, respectively. The observed *Aspergillus* colony counts from the sampled air at each location are presented in Fig. 2. While there were seasonal variations in the number of identified *Aspergillus* species, no significant correlation was found between observed fungal CFU and seasonality and/or outdoor humidity. No statistical correlation between the monthly development of culture-positive proven/probable IA and number of environmental *Aspergillus* isolates identified from air sampling was found (Spearman’s rho = –0.049, *P* = 0.887).

### Molecular and phylogenetic analysis.

Molecular identification results of clinical and environmental *Aspergillus* isolates are shown in Table 1. Cryptic *Aspergillus* species accounted for 33.8% of all isolates. There was no difference in the proportion of cryptic species among the following three groups: clinical isolates considered pathogens (30.5%), all clinical isolates (30.4%), and environmental isolates (34.1%). In the *Aspergillus* section *Fumigati* (*n* = 95), four cryptic species (4.2%) were identified; A. lentulus (*n* = 2), A. udagawae (*n* = 1), and A. turcosus (*n* = 1). Unlike *Aspergillus* section *Fumigati*, section *Nigri* presented a high proportion (70.5%) of cryptic species, mainly attributed to A. tubingensis and A. awamori. A. tubingensis was common in the environment (23.3% [7 of 30] versus 76.7% [23 of 30], *P* = 0.004), while *A. awamori* (60.7% [17 of 28] versus 39.3% [11 of 28], *P* = 0.007) was significantly prevalent in clinical isolates. In other sections, *A. persii* (*n* = 1) in section *Circumdati*, *A. niveus* (*n* = 1) in section *Terrei*, and A. sydowii (*n* = 10) in section *Versicolores* were identified (Table 1).

**TABLE 1:** *Aspergillus* isolates identified by gene sequencing in cryptic species level^*a*^

Subgenus	Section	Species	No. of isolates			
			Clinical, pathogens only (*n* = 82)	Clinical, all (*n* = 102)	Environmental (*n* = 129)	Total (*n* = 231)
*Fumigati*	*Fumigati*	A. fumigatus	38	45	46	91
		A. lentulus*	1	2	0	2
		A. udagawae*	1	1	0	1
		A. turcosus*	0	0	1	1
*Circumdati*	*Nigri*	A. niger	0	1	25	26
		A. tubingensis*	5	7	23	30
		A. awamori*	14	17	11	28
		A. acidus*	1	2	2	4
	*Flavi*	A. flavus	12	15	7	22
	*Circumdati*	A. persii*	1	1	0	1
*Terrei*	*Terrei*	A. terreus	5	5	1	6
		A. niveus*	0	1	0	1
*Nidulantes*	*Nidulantes*	A. nidulans	2	2	6	8
	*Vesicolores*	A. sydowii*	2	3	7	10
No. of cryptic *Aspergillus* spp.			25	34	44	78
% cryptic *Aspergillus* spp.			30.5	30.4	34.1	33.8

a *, Cryptic *Aspergillus* spp.

**FIG 3:**
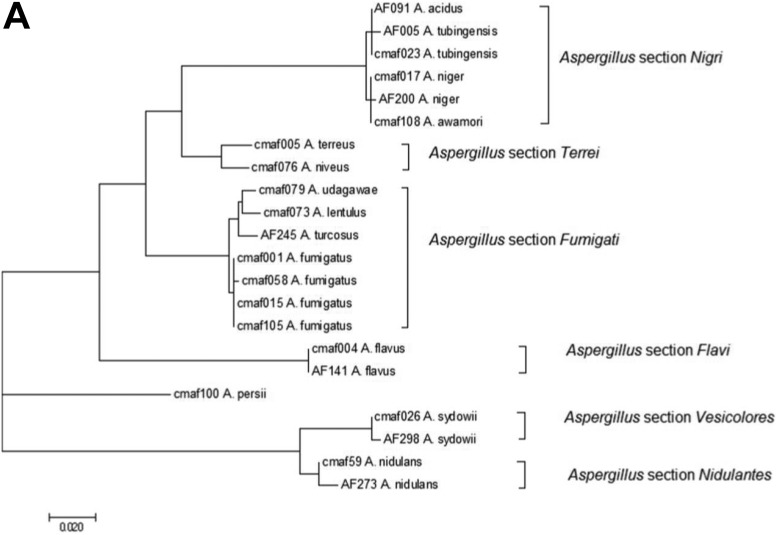

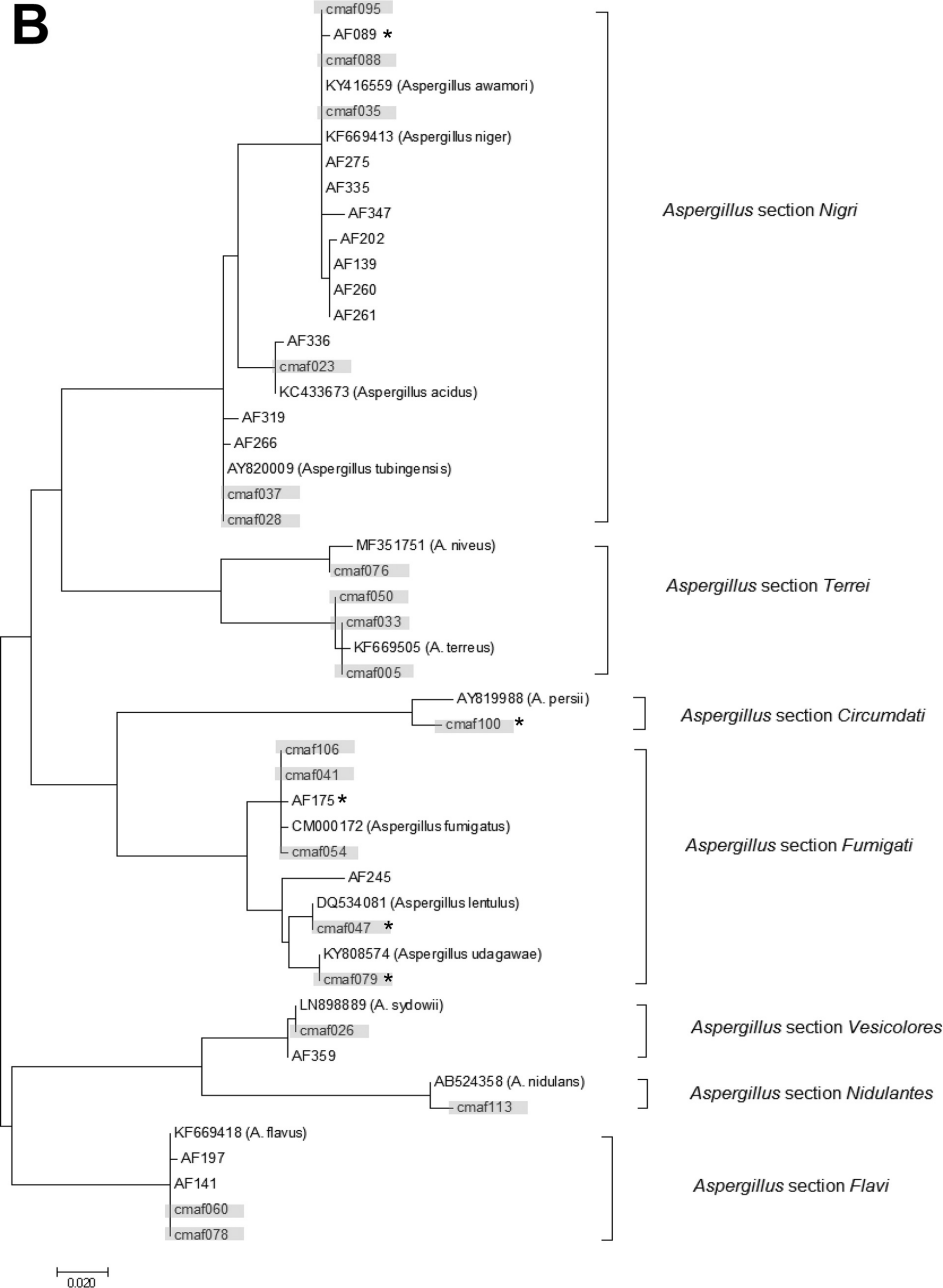
Molecular phylogenetic analysis using ITS (A) and *benA* (B) sequencing of representative isolates of *Aspergillus* by the maximum-likelihood method. Clinical isolates are presented in gray shades, and resistant isolates are marked with an asterisk.

Phylogenetic analysis using ITS and *benA* sequencing for representative isolates of *Aspergillus* is presented in Fig. 3. Clinical *Aspergillus* isolates (presented in gray shades) were closely related to environmental isolates of each section, and isolates with elevated MICs (presented with asterisk) also closely related to susceptible isolates (Fig. 3B). The phylogenetic analysis of *cyp51A* also showed that clinical and environmental A. fumigatus isolates are closely related (see Fig. SA in the supplemental material). Molecular phylogenetic tree of *benA* and *cyp51A* for *Aspergillus* section *Nigri* presented four clades and two groups: A. awamori/A. niger and A. acidus/A. tubingensis (see Fig. SB in the supplemental material). Although clinical and environmental isolates coexisted, clinical isolates were predominant in the *A. awamori* clade, and environmental isolates were predominant in the A. niger clade.

### Susceptibility of *Aspergillus* isolates.

The MIC and MEC data of four common *Aspergillus* sections are presented in Table 2. In *Aspergillus* section *Fumigati*, the MIC_90_ values were 0.5, 1, 0.25, and 1 μg/ml for itraconazole, voriconazole, posaconazole, and AMB, respectively. In *Aspergillus* section *Nigri*, the MIC_90_ values for itraconazole, voriconazole, posaconazole, and AMB were 4, 4, 0.5, and 0.5 μg/ml, respectively. In *Aspergillus* section *Flavi* and *Terrei*, the MIC_90_ values for AMB were 2 and 4 μg/ml, respectively, whereas the MICs for azoles were favorable (Table 2). The MEC_90_ for echinocandins was ≤0.25 μg/ml for the *Aspergillus* isolates in this study.

**TABLE 2:** Geometric mean, range, modal MIC, MIC_50_, and MIC_90_ in four common *Aspergillus* sections^*a*^

Section (no. of isolates)	Parameter	MIC (μg/ml)				MEC (μg/ml)		
		ITC	VRC	PSC	AMB	CAS	MICA	ANID
*Fumigati* (95)	GM	0.230	0.230	0.124	0.525	0.082	0.083	0.074
	Modal MIC	0.25	0.25	0.125	1	0.06	0.06	0.06
	MIC_50/90_	0.25/0.5	0.25/1	0.125/0.25	0.5/1	0.06/0.25	0.06/0.25	0.06/0.125
	Range	0.06–64	0.06–64	0.06–2	0.06–16	0.06–2	0.06–0.25	0.06–1
*Nigri* (88)	GM	0.867	0.886	0.260	0.228	0.073	0.083	0.063
	Modal MIC	0.5	1	0.25	0.25	0.06	0.06	0.06
	MIC_50/90_	0.5/4	1/4	0.25/0.5	0.25/0.5	0.06/0.06	0.06/0.25	0.06/0.06
	Range	0.125–16	0.06–64	0.06–1	0.06–8	0.06–8	0.06–0.25	0.06–0.25
*Flavi* (22)	GM	0.293	0.882	0.133	1.370	0.144	0.118	0.088
	Modal MIC	0.25	1	0.125	1–2^*b*^	0.06	0.06	0.06
	MIC_50/90_	0.25/0.5	1/1	0.125/0.125	1/2	0.125/0.25	0.06/0.25	0.06/0.125
	Range	0.25–0.5	0.25–32	0.06–1	0.25–4	0.06–4	0.06–0.25	0.06–0.125
*Terrei* (7)	GM	0.25	0.552	0.082	1.516	0.082	0.067	0.06
	Modal MIC	0.25	0.5	0.06	1	0.06	0.06	0.06
	MIC_50/90_	0.25/0.5	0.5/1	0.06/0.125	1/4	0.06/0.25	0.06/0.125	0.06/0.06
	Range	0.125–0.5	0.25–1	0.06–0.125	1–4	0.06–0.25	0.06–0.125	0.06–0.06

a AMB, amphotericin B; ANID, anidulafungin; CAS, caspofungin; GM, geometric mean; ITC, itraconazole; MEC, minimal effective concentration; MICA, micafungin; PSC, posaconazole; VRC, voriconazole.

b Identical numbers of isolates (*n* = 10) had MICs of 1 and 2 μg/ml.

In noncryptic species, we evaluated the resistance rate according to the CLSI ECV, as suggested in Materials and Methods. The resistance rate of A. fumigatus (*n* = 91) was 6.6% (*n* = 6) against any azole and 2.2% (*n* = 2) against AMB. In A. niger (*n* = 26), the resistance rate was 3.8% (*n* = 1) against any azole and 3.8% (*n* = 1) against AMB. Similarly, the azole resistance rate of A. flavus was 4.8% (*n* = 1). The resistant A. flavus originated from the environment (outdoor air), which showed high MICs against voriconazole (32 μg/ml) and posaconazole (1 μg/ml). In *cyp51* gene sequencing, S263L mutation in *cyp51A* and M54T, S240A, D254G, and N423D mutation in *cyp51C* were found (see Table SB in the supplemental material). However, the same sequence group (sequence group K) was found in susceptible A. flavus environmental isolates.

The MIC distributions of cryptic species are presented in Table 3. Isolates of cryptic species showed distinct susceptibility patterns. Although A. lentulus showed elevated MIC for AMB (MIC = 4 μg/ml), *A. udagawae* and *A. turcosus* showed favorable MIC results (Table 3). In A. tubingensis, azole susceptibility was relatively higher, with MIC_50/90_s of 2/8, 2/4, and 0.5/0.5 μg/ml for itraconazole, voriconazole, and posaconazole, respectively. However, *A. awamori* showed lower MICs compared to A. tubingensis: MIC_50/90_s of 0.5/1 μg/ml for itraconazole and voriconazole and 0.25/0.25 μg/ml for posaconazole. A. subramanianii revealed the highest MIC (64 μg/ml) against AMB.

**TABLE 3:** MIC distribution of cryptic *Aspergillus* species^*a*^

Section	Species (*n*)	Antifungal agents	MIC/MEC (μg/ml)										
			0.06	0.125	0.25	0.5	1	2	4	8	16	32	64
*Fumigati*	A. lentulus (2)	ITC			1	1							
		VRC					2						
		PSC		1			1						
		AMB							2				
		CAS	1				1						
		MICA	1	1									
		ANID	2										
	A. udagawae (2)	ITC			1								
		VRC				1							
		PSC			1								
		AMB				1							
		CAS	1										
		MICA	1										
		ANID	1										
	A. turcosus (1)	ITC		1									
		VRC		1									
		PSC	1										
		AMB				1							
		CAS	1										
		MICA	1										
		ANID	1										
*Nigri*	A. tubingensis (30)	ITC			1	1	5	14	4	4	1		
		VRC		1	1		4	17	6				1
		PSC		1	9	19	1						
		AMB	8	2	14	5	1						
		CAS	26		1				1	1			
		MICA	17	5	8								
		ANID	28	1	1								
	A. awamori (28)	ITC			5	16	5	2					
		VRC	2	1	6	13	4	2					
		PSC		10	17	1							
		AMB	5	3	7	8	4	1					
		CAS	26		1	1							
		MICA	24	3	1								
		ANID	27	1									
	A. acidus (4)	ITC			1		3						
		VRC			1		2	1					
		PSC			3	1							
		AMB			1	3							
		CAS	3	1									
		MICA	2	1	1								
		ANID	3	1									
*Circumdati*	A. persii (1)	ITC						1					
		VRC						1					
		PSC				1							
		AMB											1
		CAS	1										
		MICA	1										
		ANID	1										
*Terrei*	A. niveus (1)	ITC			1								
		VRC					1						
		PSC		1									
		AMB						1					
		CAS	1										
		MICA	1										
		ANID	1										
*Vesicolores*	A. sydowii (10)	ITC		1	3	6							
		VRC			4	3	3						
		PSC	2	5	3								
		AMB			1	2	4	3					
		CAS	8			3							
		MICA	4	3	3								
		ANID	8	2									

a AMB, amphotericin B deoxycholate; ANID, anidulafungin; CAS, caspofungin; ITC, itraconazole; MEC, minimal effective concentration; MICA, micafungin; PSC, posaconazole; VRC, voriconazole.

### *cyp51A* mutation of *A. fumigatus* and clinical outcome.

The azole susceptibility and *cyp51A* mutation results for A. fumigatus, along with clinical information, are presented in Table 4. The resistance rates of A. fumigatus to any azole were 5.3% (*n* = 2), 6.7% (*n* = 3), and 6.5% (*n* = 3) in pathogenic clinical isolates (*n* = 38), all clinical isolates regardless of clinical significance (*n* = 45), and environmental isolates (*n* = 46), respectively. The azole resistance rates were not significantly different among the three groups. (5.3% [2 of 38], 6.7% [3 of 45], and 6.5% [3 of 46], *P* = 0.667). Among the six azole-resistant A. fumigatus (ARAF) isolates, there were two itraconazole and posaconazole cross-resistant isolates and one pan-azole-resistant isolate (Table 4). These three isolates had mutations that included TR_34_/L98H in the *cyp51A* gene. The pan-azole-resistant strain harbored TR_34_/L98H with T289A, I364V, and G448S mutations. In addition, two mono-azole-resistant isolates revealed an elevated MIC (1 μg/ml) against posaconazole; none of these isolates were pathogenic, and no *cyp51A* mutations, including TR_34_/L98H, were observed. The other isolate revealed elevated MICs to voriconazole (8 μg/ml) and posaconazole (0.5 μg/ml), which originated from the air collected outside the hospital, and no *cyp51A* mutation was confirmed. Several polymorphisms, including F92L, Y121D, and N248K, which did not present significant clinical implications, were found in eight susceptible isolates.

**TABLE 4:** Azole susceptibilities, tandem repeat in the promoter, and *cyp51A* mutation in Aspergillus fumigatus isolates^*a*^

Source and azole susceptibility	TR	*cyp51A* mutation (*n*)	MIC (μg/ml)^*b*^			Azole exposure (no. of days)^*c*^	Tx	Outcome	
			ITC	VRC	PSC			IA-related death	100-day overall death
Clinical, pathogens only (*n* = 38)									
Susceptible (*n* = 36)	(–)^*d*^	None (28)	0.125–1	0.06–1	0.06–0.25			10/28	12/28
	(–)	F92L	0.5	0.5	0.125	None	VRC	FU loss	FU loss
	(–)	F92L	0.25	0.5	0.125	None	None	No	Survival
	(–)	L375S	0.25	0.25	0.06	None	L-AMB→VRC	No	Survival
	(–)	N248K	0.5	0.25	0.06	ITC (104)	VRC	No	Survival
	(–)	Y121D, N248K	0.5	0.25	0.125	None	VRC	No	Survival
	(–)	Y121D	0.5	0.25	0.06	None	L-AMB	Yes	Death
	(–)	F92L, Y121D, E180D	1	0.5	0.06	None	VRC	No	Death
	(–)	Q249K	0.5	1	0.125	None	VRC	Yes	Death
Resistant (*n* = 2)	34	L98H, S297T, F495I	≥64	1	4	PSC (87)	VRC	No	Death
	34	L98H, T289A, I364V, G448S	2	≥64	0.5	PSC (56)	L-AMB	Yes	Death
									
Clinical, nonpathogens (*n* = 7)									
Susceptible (*n* = 6)	(–)	None (6)	0.25	0.06–0.5	0.06–0.25		None	NA	1/6
Resistant (*n* = 1)	(–)	None (1)	0.25	0.125	1	None	None	No	Survival
									
Environment (*n* = 46)									
Susceptible (*n* = 43)	(–)	None (43)	0.06–0.5	0.06–1	0.06–0.25	NA	NA	NA	NA
Resistant (*n* = 3)	(–)	None (1)	0.06	0.25	1	NA	NA	NA	NA
	(–)	None (1)	0.25	8	0.5	NA	NA	NA	NA
	34	L98H, Q193P	2	1	0.5	NA	NA	NA	NA

a FU, follow-up; IA, invasive aspergillosis; ITC, itraconazole; L-AMB, liposomal amphotericin B; NA, not applicable; PSC, posaconazole; TR, tandem repeat; Tx, treatment; VRC, voriconazole.

b The MIC value is presented with the range if more than two isolates were applicable.

c This value only includes exposure of mold active azole prior to the diagnosis of proven/probable invasive aspergillosis.

d “(–)” means that no TR was found.

We divided proven/probable IA cases caused by A. fumigatus into azole-susceptible (*n* = 36) and azole-resistant (*n* = 2) groups (Table 4). IA-related mortality rates were 33.3% versus 50% in the azole-susceptible and azole-resistant groups (*P* = 0.573), respectively, while the overall mortality at 100 days was 41.7% versus 100% (*P* = 0.193).

## DISCUSSION

We identified the distribution and susceptibility of *Aspergillus* isolates and compared the differences among the groups of pathogens, all clinical isolates, and environmental isolates. Among the three groups, the proportion of cryptic *Aspergillus* species was not different, and the azole resistance rates for A. fumigatus were also similar between clinical and environmental isolates. The incidence of proven/probable IA was 1.3 cases/1,000 patient-days, without significant correlation between the development of IA and monthly environmental fungal density from air sampling. Thus, host factors, as well as environmental factors, are important for IA development.

In section *Fumigati*, 4.2% of *Aspergillus* isolates were cryptic species. A. lentulus was characterized with high MICs for AMB, which is in line with previous studies. *A. udagawae* and *A. turcosus* showed favorable MICs (<0.5 μg/ml) for all azoles, AMB, and echinocandins. The cryptic species detected here mainly comprised A. tubingensis and *A. awamori* in section *Nigri*. A. tubingensis was more common among environmental isolates, while *A. awamori* was common among clinical isolates. Frequently, isolates identified as A. niger by ITS sequencing turned up as *A. awamori* by *benA* sequencing, accounting for 82% of the section *Nigri* clinical isolates. This finding is consistent with a report of *A. awamori* being one of the common cryptic species (20, 26). Fortunately, *A. awamori* showed a more favorable MIC than that of A. tubingensis (voriconazole MIC_90_: 1 μg/ml versus 4 μg/ml). However, it is necessary to establish a MIC breakpoint that can reflect the epidemiology and treatment response based on further data collection, given that *A. awamori* is one of the major cryptic species in clinical pathogenic isolates.

We found the azole resistance rate of A. fumigatus to be 5.3% in pathogenic isolates from hematologic malignancy patients with IA, similar to that of environmental isolates (6.5%). The azole resistance rate in A. fumigatus was not significantly different among pathogens, clinical isolates regardless of clinical significance, and environmental isolates from air sampling. In some Dutch regions, the environmental azole resistance rate is known to exceed 10%. Another UK study reported an environmental azole resistance rate of 6.0%, and most of these isolates harbored the TR_34_/L98H mutation (27). Although Catholic Hematology Hospital is located in Seoul, a highly urbanized city in South Korea, the results of this study might also reflect environmental epidemiology, implicating effects of the agricultural areas in South Korea, considering that fungal spores can travel up to 1,500 km (28). Crop protection chemicals can affect the azole resistance rate in environmental fungi (12). The pesticide consumption in Korea is reported to be 6 kg/ha, similar to that in the United States (6 kg/ha), but is less than that of Japan (12 kg/ha) and Taiwan (16 kg/ha) (29).

Since Denning et al. reported the relationship between azole resistance and poor survival rate in a neutropenic murine model in 1997, azole resistance has been observed to be increasing in clinical A. fumigatus isolates (30–32). In 1997, three itraconazole-resistant A. fumigatus isolates were identified in the Netherlands (31). Two years later, four itraconazole-resistant isolates were reported from France (32). The global azole resistance rate of A. fumigatus has recently been reported to be 2 to 31% (10, 33–40). Azole resistance rates vary according to the study period, region, the inclusion of MIC for itraconazole only or not, and patient type (hematologic malignancies only or chronic lung diseases such as cystic fibrosis with aspergilloma), etc. A study performed in the United States reported an increase in azole resistance in the period from 2003 to 2015 from that in 1999 to 2002 (14).

ARAF with the TR_34_/L98H mutation have showed poor clinical outcome, with a 12-week mortality rate of 88% (33). Another study from Germany on HSCT recipients also reported 100% mortality 100 days after the detection of ARAF (39). Our data also revealed a high mortality rate in the azole-resistant group, with an IA-related mortality of 50% and 100-day all-cause mortality of 100%. Although the outcome of IA seems to have improved in recent prospective trials, real-world data continue to show high mortality: approximately 30 to 60% at 90 days (3, 38–44). Considering that IA-related deaths in the azole-susceptible group comprised 33% in this study, the mortality in hematology patients with IA caused by ARAF was higher than that in overall IA cases. Although this is not representative of mortality data in South Korea because there were only two ARAF IA cases, clinicians should note that 100-day all-cause mortality in the resistant group was 100% in this study. However, in another patient cohort, no correlation between *in vitro* MICs and 42-day mortality in aspergillosis patients was found (14). Nevertheless, high MICs and TR_34_/L98H *cyp51A* mutations are significant factors in most studies. The relationship between increased azole MICs and treatment failure of IA needs to be understood and analyzed to finally determine the future breakpoint of azole MIC. Furthermore, in this study, ARAF pathogenic isolates was identified from patients with prior azole use. It is not possible to interpret the association at present, and more cases should be collected for ARAF developed during azole exposure.

Based on the studies reported so far, there are data on the resistance phenotype of ARAF, which is known to be more relevant when certain *cyp51A* gene mutations are present: TR_34_/L98H can reveal pan-azole resistance, and TR_46_/Y121F/T289A is usually related to voriconazole resistance with variable susceptibility to posaconazole. G448S substitution is known to be associated with voriconazole resistance (susceptibility to itraconazole and posaconazole). Interestingly, one pan-azole-resistant A. fumigatus isolate in this study harbored TR_34_/L98H/T289A/I354V/G448S, but it did not harbor Y121F. This result implies that various mutations can be combined, which make it difficult to explain all the azole resistance phenotypes using current knowledge.

We also identified resistance for non-*fumigatus Aspergillus* and sequencing of *cyp51A*, *cyp51B*, and *cyp51C* for A. flavus. The resistance rate of common non-*fumigatus Aspergillus* was <5% according to the CLSI ECV. *cyp51C* revealed genotype diversity and various mutations, which are classified into 14 sequence groups. As is known, *cyp51C* is more polymorphic than ITS, *benA*, or *cyp51A* for A. flavus. One resistant A. flavus isolate was identified from air collected outside the hospital building. Its sequence group was also found in susceptible A. flavus environmental isolates. Further studies are needed to determine the mechanism of azole-resistant A. flavus.

The strengths of this study are manifold. First, we distinguished clinical *Aspergillus* isolates into pathogenic and colonization typee and included environmental isolates collected throughout a year during the study period. Second, our study included the clinical outcome of patients with IA caused by ARAF. Third, we focused only on culture-positive proven/probable IA in patients with hematologic malignancies, which is a homogeneous group.

The major results of this study can be summed up as follows. First, cryptic species comprised one-third of all *Aspergillus* isolates, with no difference in the rate between clinical and environmental isolates. Second, A. tubingensis occurred with a higher dominance in environmental samples, while *A. awamori* was more common in clinical isolates. Third, ARAF was found in 5.3% of all A. fumigatus isolates. The azole resistance rate was not different between clinical and environmental isolates. Fourth, patients with IA caused by ARAF presented poor outcome in the aspect of all-cause 100-day mortality. Insights from this study can contribute to epidemiology data accumulation and therapeutic advances in the future.

## Supplementary Material

Supplemental file 1
