# Supplementary material for: Epidemiology and Antifungal Susceptibility Profile of Aspergillus Species: Comparison between Environmental and Clinical Isolates from Patients with Hematologic Malignancies
Source: J Clin Microbiol. 2019 Jun 25;57(7):e02023-18. doi: 10.1128/JCM.02023-18 (PMC6595445; doi:10.1128/JCM.02023-18)
Supplement: Supplemental file 1 [file JCM.02023-18-s0001.pdf]

Appendixes

Appendix A. Control sequences used in this study

The following control sequences were used: *BenA* of CM000172 (*A. fumigatus*), DQ534081 (*A. lentulus*), KY808574 (*A. udagawae*), KY808577 (*A. wyomingensis*), KY808599 (*A. frankstonensis*), GQ376143 (*A. fumigatiaffinis*), KC433673 (*A. acidus*), KY416559 (*A. awamori*), AY820013 (*A. piperis*), KF669413 (*A. niger*), and AY820009 (*A. tubingensis*), *cyp51A* of CM000172 (azole-susceptible *A. fumigatus*), KP270710 (azole-resistant *A. fumigatus*), GU479991 (*A. lentulus*), JF450900 (*A. niger*), JF450904 (*A. niger*), JF450905 (*A. tubingensis*), JF450906 (*A. awamori*), and JF450909 (*A. acidus*).

Table A. Epidemiological cutoff values applied for common *Aspergillus* species in this study

species	MIC/MEC breakpoints (µg/mL)				
	**ITC**	**VRC**	**PSC**	**CAS**	**AMB**
A. fumigatus	1	1	0.25	0.5	2
A. niger	4	2	2	0.5	2
A. flavus	1	2	0.5	0.12	4
A. terreus	2	2	1	0.12	4

Abbreviations. AMB, amphotericin B deoxycholate; CAS, caspofungin; ITC, itraconazole; MEC, minimal effective concentration; MIC, minimal inhibitory concentration; PSC, posaconazole; VRC, voriconazole

Reference.

1) Clinical and Laboratory Standards Institute. 2016. Epidemiological Cutoff Values for Antifungal Susceptibility Testing. 1st Ed. CLSI supplement M59. CLSI, Wayne, PA, USA.,
2) Espinel-Ingroff A, Turnidge J, Alastruey-Izquierdo A, Dannaoui E, Garcia-Effron G, Guinea J, Kidd S, Pelaez T, Sanguinetti M, Meletiadis J, Botterel F, Bustamante B, Chen YC, Chakrabarti A, Chowdhary A, Chryssanthou E, Córdoba S, Gonzalez GM, Guarro J, Johnson EM, Kus JV, Lass-Flörl C, Linares-Sicilia MJ, Martín-Mazuelos E, Negri CE, Pfaller MA, Tortorano AM. 2018. Posaconazole MIC Distributions for *Aspergillus fumigatus* Species Complex by Four Methods: Impact of *cyp51A* Mutations on Estimation of Epidemiological Cutoff Values. Antimicrob Agents Chemother 62:e01916-17. https://doi.org/10.1128/AAC.01916-17.

Table B. Analysis of *cyp51s* mutation in *Aspergillus flavus* clinical and environmental isolates.

**Azole susceptibility**	***cyp51 mutations***			***cyp51s* sequence group**	**no. of isolates**		**Representative of Strain**
	cyp51A	***cyp51B***	***cyp51C***		**Clinical isolates (n)**	**Environmental isolates (n)**	
Susceptible (n = 21)	None	None	M54T, S240A	A	1	0	cmaf074
	None	None	M54T, S240A, D254N, I284V	B	2	0	cmaf049
	None	None	M54T, S240A, S399I	C	0	1	AF164
	None	None	M54T, S240A, D254G, N423D	D	1	0	cmaf094
	None	None	M54T, S240A, P419T, N423D	E	2	0	cmaf060
	None	None	T34A, M54T, S240A, D254G, N423D	F	2	1	cmaf004
	A205T	None	M54T, S240A	G	0	2	AF280
	A205T	None	M54T, S240A, D254N, I284V	H	1	0	cmaf091
	D213N	None	M54T, S240A	I	2	0	cmaf099
	D213N	K165E	M54T, S240A	J	1	0	cmaf107
	S263L	None	M54T, S240A, D254G, N423D	K	0	1	AF197
	R242C	None	M54T, S240A, A324T	L	1	0	cmaf018
	None	K165E	N/A*	M	2	1	cmaf003
	T335A	None	M54T, S240A	N	-	-	ATCC16883†
Resistant (n = 1)	S263L	None	M54T, S240A, D254G, N423D	K	0	1	AF334

**cyp51C* was not sequenced in three *A. flavus* isolates in this study, which need to be further studied.
†reference strain

Figure A. Molecular phylogenetic analysis of the *benA* (a) and *cyp51A* (b) for the clinical and environmental the *A. fumigatus* isolates. Clinical isolates are presented in gray shades, and resistant isolates are marked with an asterisk.

Figure B. Molecular phylogenetic analysis of the ITS (a), *benA* (b) and *cyp51A* (c) of *Aspergillus* section *Nigri* isolates by maximum likelihood method. Clinical isolates are presented in gray shades.

Figure A (a)

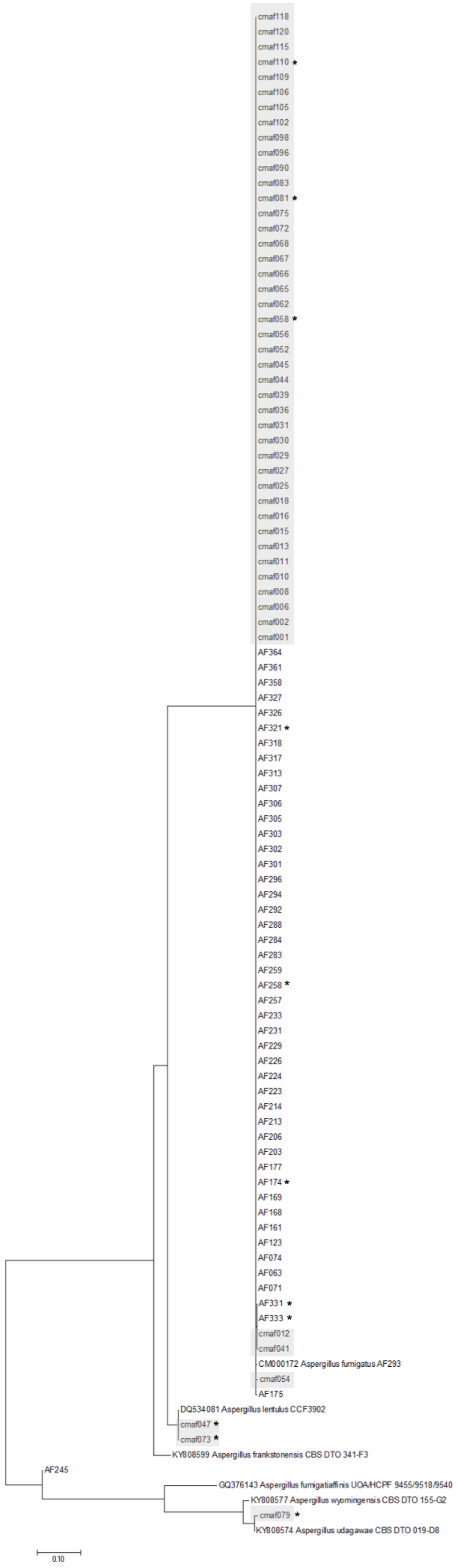

cmaf118
cmaf120
cmaf115
cmaf110 *
cmaf109
cmaf106
cmaf105
cmaf102
cmaf098
cmaf096
cmaf090
cmaf083
cmaf081 *
cmaf075
cmaf072
cmaf068
cmaf067
cmaf066
cmaf065
cmaf062
cmaf058 *
cmaf056
cmaf052
cmaf045
cmaf044
cmaf039
cmaf036
cmaf031
cmaf030
cmaf029
cmaf027
cmaf025
cmaf018
cmaf016
cmaf015
cmaf013
cmaf011
cmaf010
cmaf008
cmaf006
cmaf002
cmaf001
AF364
AF361
AF358
AF327
AF326
AF321 *
AF318
AF317
AF313
AF307
AF306
AF305
AF303
AF302
AF301
AF296
AF294
AF292
AF288
AF284
AF283
AF259
AF258 *
AF257
AF233
AF231
AF229
AF226
AF224
AF223
AF214
AF213
AF206
AF203
AF177
AF174 *
AF169
AF168
AF161
AF123
AF074
AF063
AF071
AF331 *
AF333 *
cmaf012
cmaf041
CM000172 Aspergillus fumigatus AF293
cmaf054
AF175
DQ534081 Aspergillus lentulus CCF3902
cmaf047 *
cmaf073 *
KY808599 Aspergillus frankstonensis CBS DTO 341-F3
AF245
GQ376143 Aspergillus fumigatiaffinis UOA/HCPF 9455/9518/9540
KY808577 Aspergillus wyomingensis CBS DTO 155-G2
cmaf079 *
KY808574 Aspergillus udagawae CBS DTO 019-D8
0.10

Figure A (b)

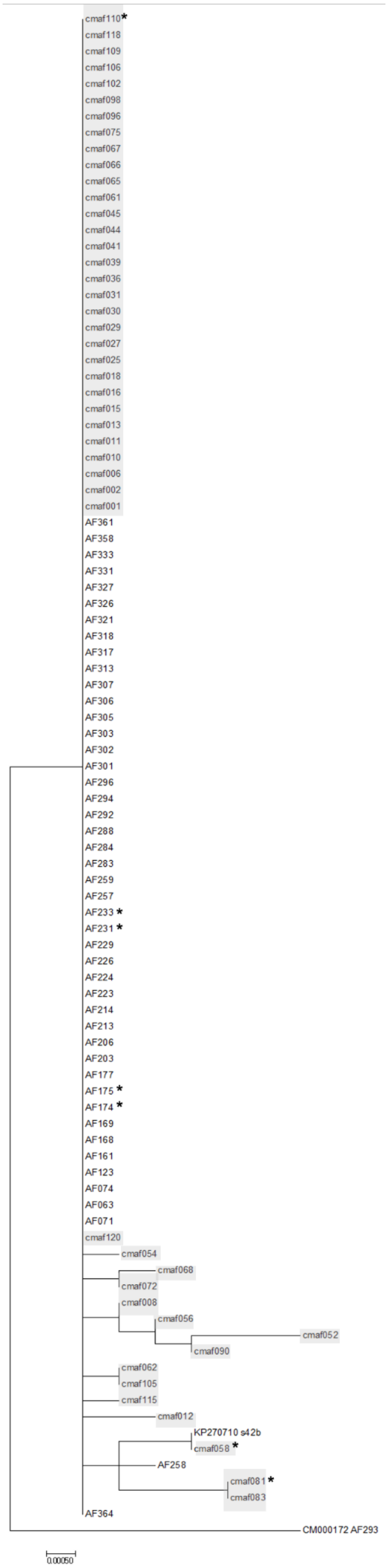
cmaf110*
cmaf118
cmaf109
cmaf106
cmaf102
cmaf098
cmaf096
cmaf075
cmaf067
cmaf066
cmaf065
cmaf061
cmaf045
cmaf044
cmaf041
cmaf039
cmaf036
cmaf031
cmaf030
cmaf029
cmaf027
cmaf025
cmaf018
cmaf016
cmaf015
cmaf013
cmaf011
cmaf010
cmaf006
cmaf002
cmaf001
AF361
AF358
AF333
AF331
AF327
AF326
AF321
AF318
AF317
AF313
AF307
AF306
AF305
AF303
AF302
AF301
AF296
AF294
AF292
AF288
AF284
AF283
AF259
AF257
AF233*
AF231*
AF229
AF226
AF224
AF223
AF214
AF213
AF206
AF203
AF177
AF175*
AF174*
AF169
AF168
AF161
AF123
AF074
AF063
AF071
cmaf120
cmaf054
cmaf068
cmaf072
cmaf008
cmaf056
cmaf052
cmaf090
cmaf062
cmaf105
cmaf115
cmaf012
KP270710 s42b
cmaf058*
AF258
cmaf081*
cmaf083
AF364
CM000172 AF293
0.00050

Figure B (a)

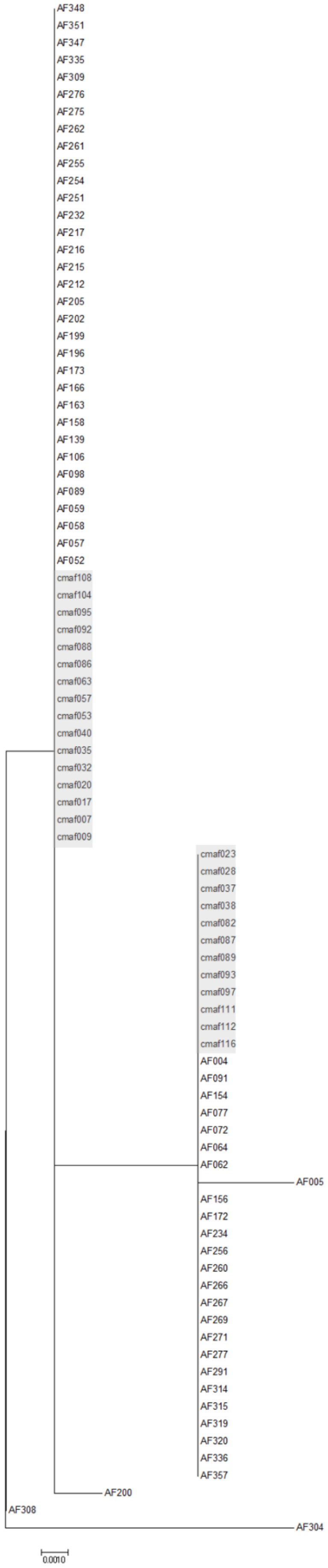
AF348
AF351
AF347
AF335
AF309
AF276
AF275
AF262
AF261
AF255
AF254
AF251
AF232
AF217
AF216
AF215
AF212
AF205
AF202
AF199
AF196
AF173
AF166
AF163
AF158
AF139
AF106
AF098
AF089
AF059
AF058
AF057
AF052
cmaf108
cmaf104
cmaf095
cmaf092
cmaf088
cmaf086
cmaf063
cmaf057
cmaf053
cmaf040
cmaf035
cmaf032
cmaf020
cmaf017
cmaf007
cmaf009
cmaf023
cmaf028
cmaf037
cmaf038
cmaf082
cmaf087
cmaf089
cmaf093
cmaf097
cmaf111
cmaf112
cmaf116
AF004
AF091
AF154
AF077
AF072
AF064
AF062
AF005
AF156
AF172
AF234
AF256
AF260
AF266
AF267
AF269
AF271
AF277
AF291
AF314
AF315
AF319
AF320
AF336
AF357
AF200
AF308
AF304
0.0010

Figure B (b)

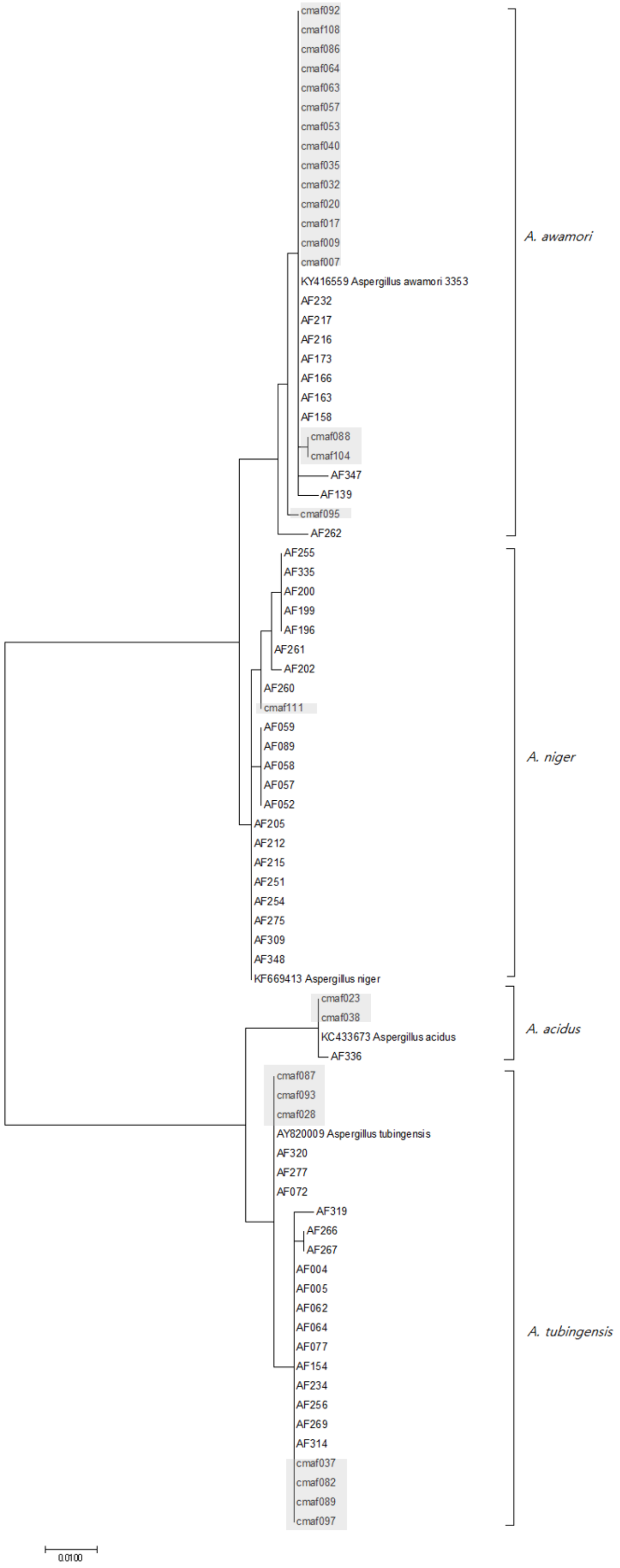
cmaf092
cmaf108
cmaf086
cmaf064
cmaf063
cmaf057
cmaf053
cmaf040
cmaf035
cmaf032
cmaf020
cmaf017
cmaf009
cmaf007
KY416559 Aspergillus awamori 3353
AF232
AF217
AF216
AF173
AF166
AF163
AF158
cmaf088
cmaf104
AF347
AF139
cmaf095
AF262
A. awamori
AF255
AF335
AF200
AF199
AF196
AF261
AF202
AF260
cmaf111
AF059
AF089
AF058
AF057
AF052
AF205
AF212
AF215
AF251
AF254
AF275
AF309
AF348
KF669413 Aspergillus niger
A. niger
cmaf023
cmaf038
KC433673 Aspergillus acidus
AF336
A. acidus
cmaf087
cmaf093
cmaf028
AY820009 Aspergillus tubingensis
AF320
AF277
AF072
AF319
AF266
AF267
AF004
AF005
AF062
AF064
AF077
AF154
AF234
AF256
AF269
AF314
cmaf037
cmaf082
cmaf089
cmaf097
A. tubingensis
0.0100

Figure B (c)

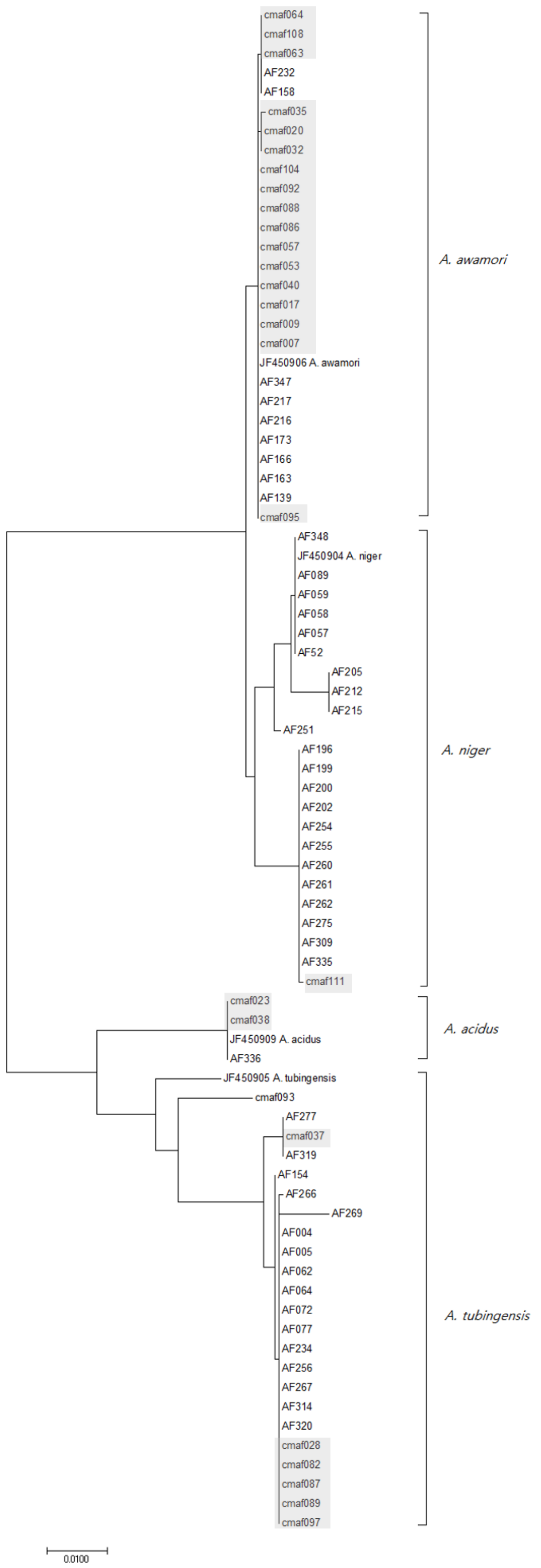
cmaf064
cmaf108
cmaf063
AF232
AF158
cmaf035
cmaf020
cmaf032
cmaf104
cmaf092
cmaf088
cmaf086
cmaf057
cmaf053
cmaf040
cmaf017
cmaf009
cmaf007
JF450906 A. awamori
AF347
AF217
AF216
AF173
AF166
AF163
AF139
cmaf095
A. awamori
AF348
JF450904 A. niger
AF089
AF059
AF058
AF057
AF52
AF205
AF212
AF215
AF251
AF196
AF199
AF200
AF202
AF254
AF255
AF260
AF261
AF262
AF275
AF309
AF335
cmaf111
A. niger
cmaf023
cmaf038
JF450909 A. acidus
AF336
A. acidus
JF450905 A. tubingensis
cmaf093
AF277
cmaf037
AF319
AF154
AF266
AF269
AF004
AF005
AF062
AF064
AF072
AF077
AF234
AF256
AF267
AF314
AF320
cmaf028
cmaf082
cmaf087
cmaf089
cmaf097
A. tubingensis
0.0100
